# The Left Atrial Appendage and Atrial Fibrillation—A Contemporary Review

**DOI:** 10.3390/jcm12216909

**Published:** 2023-11-02

**Authors:** Ralf Martz Sulague, Tarik Whitham, Lester Mico Lopez Danganan, Victory Effiom, Katherine Candelario, Nida Latif, Irbaz Hameed

**Affiliations:** 1 Graduate School of Arts and Sciences, Georgetown University, Washington, DC 20057, USA; rs2141@georgetown.com; 2 College of Medicine, Northeast Ohio Medical University, Rootstown, OH 44272, USA; twitham@neomed.cedu; 3 Faculty of Medicine and Surgery, University of Santo Tomas, Sampaloc, Manila 1008, Philippines; dangananlester@gmail.com; 4 College of Medical Sciences, University of Calabar, Calabar 540271, Nigeria; veffiom24@gmail.com; 5 Division of Cardiac Surgery, Department of Surgery, Yale School of Medicine, 330 Cedar Street, New Haven, CT 06510, USA; katherinemarinac@gmail.com (K.C.); nida.latif268@gmail.com (N.L.)

**Keywords:** left atrial appendage, left atrial appendage occlusion, atrial fibrillation

## Abstract

In patients with atrial fibrillation, the left atrial appendage may serve as the site of thrombus formation due to stasis that occurs within the appendage because of its shape and trabeculations. Although thrombus formation can be reduced by using anticoagulants, this may be contraindicated in some patients. The need for a better alternative treatment prompted the study of left atrial appendage occlusion for thromboembolism prophylaxis. Due to this, procedures that excise or occlude the left atrial appendage have gained attention because of their ability to prevent thromboembolic events. This article provides a comprehensive review of the left atrial appendage and its associated procedures’ clinical utility.

## 1. Introduction

The left atrial appendage (LAA) is an anatomical outpouching of the left atrium [1]. Found within the pericardium close to the left ventricle, it is noted to act as a decompression chamber during left ventricular systole. Such a role is influenced by its high position within the left atrium, increased distensibility, high concentrations of atrial natriuretic factor (ANF), and neuronal configuration [2].

In patients with atrial fibrillation (AF), the LAA may serve as site of thrombus formation due to the stasis that occurs within the appendage because of its shape and trabeculations. About 90% of atrial thrombi among patients with non-rheumatic atrial fibrillation and 60% among rheumatic mitral valve disease patients originate in the LAA [3]. Due to this, procedures that excise or occlude the LAA have gained attention because of their ability to prevent thromboembolic events. Furthermore, transesophageal echocardiography has offered clearer imaging of the LAA, allowing assessment of the clinical implications of the LAA based on its size, shape, content, and flow patterns [4]. Although thrombus formation can be reduced by using anticoagulants, specifically warfarin, this may be contraindicated in some patients. The need for a better alternative treatment prompted the study of LAA occlusion for thromboembolism prophylaxis [3]. This paper focuses on the anatomy, embryology, functions, imaging, and procedures involving the LAA. The objective of this paper is to provide a comprehensive review regarding the LAA and the clinical utility of its associated procedures.

## 2. Left Atrial Appendage Anatomy

### 2.1. Embryology

During the third week of gestation, the LAA begins to develop as a remnant of the embryonic left atrium, while the branches of the primordial pulmonary vein forms the remaining portion of the left atrium [5].

Following four weeks of gestation, the primitive atrium starts moving toward its ultimate location. The ensuing cellular protrusion phase solidifies the basal mesodermal layer and forms the trabeculae or pectinate muscles leading to the rough endocardium. The ultrastructural and physiological characteristics of the LAA are distinct from the left atrium [6]. 

A connection of paired cardiac mesoderm that fuses to form a two-cell thick tubular heart is where the beginning and ultimate location of the left atrial appendage and its surrounding structures originate [7].

From a pro-myocardial plate of cells that slowly forms into a three-dimensional tubular formation, it folds into an area that develops into a heart, residing in what will eventually become the pericardial space. The left atrial appendage begins to emerge as early as in these stages of embryonic growth [8].

During the fourth week of embryonic development, the structurally and physiologically distinct LAA is derived from the left wall of the primary atrium. Further development of the left atrium occurs around six weeks of embryonic life [9].

### 2.2. Anatomy

Unlike the right appendage, which is broad and triangular with a wide junction, the LAA is a finger-like, long, tubular, hooked structure, which is usually crenelated and has a narrow junction with the venous component of the atrium. The extent of the pectinate muscles enables appendages to be designated as morphologically right or left [10].

Located within the pericardium, the LAA is a blind-ended pouch emerging from the left atrium, which makes its spatial relationship of importance. The structures surrounding the LAA include (1) the superiorly directed pulmonary artery; (2) the tip of the appendage, oriented toward the left ventricle free wall pointing inferomedially; (3) the left phrenic nerve running over the appendage; (4) fibers of Bachmann’s bundle toward the LAA arising from the medial aspect of the atrial roof; (5) the left superior pulmonary vein superiorly; and (6) the mitral valve inferiorly [8].

The LAA cellular composition can vary both in composition and density since it contains both endocardial and epicardial layers that are structurally complicated by a disarray of myocyte orientation [2].

Evora et al. noted that the LAA morphology is the substrate for generating thrombi due to its direct connection to the left-sided circulation [9]. The heart is internally covered by the endocardial endothelium. These cells have a large surface area, providing a very high ratio of cavity surface area to atrial volume, which is suggestive of a significant sensory role for the endocardial endothelium [9,11].

### 2.3. Comparative Anatomy

“Lobe classification” into four types of LAA was first described by Veinot et al., in which they determined the orifice diameter, width, length, and number of lobes. The dominant type, which occurred in 54% of the hearts, is composed of two lobes, while 23% of the hearts studied have a three-lobed LAA. The third type, which occurred in 20% of the hearts, is a one-lobed appendage, while the fourth type, occurring in 3% of the examined heart, are four-lobed appendages [4]. Kaminski et al. also found the LAA orifice diameters to be sex-related. In women, they observed a smaller orifice size in all types of LAA with the biggest difference in the orifice size seen in LAA type 2, and accounting for 9.0 mm for females and 12.3 mm for males [12].

Having more complex internal characteristics, a variable number of lobes with the absence of a dominant lobe, a more irregular shape of orifice, and a short length, the “cauliflower” morphology was noted by Beigel et al. to be most often associated with an embolic event. The “cactus” has a foremost central lobe and the secondary lobes arise from it superiorly and inferiorly. The “windsock” has a dominant lobe as its primary structure with the location and number of secondary or even tertiary lobes varying. The most common morphology, “chicken wing”, has a dominant lobe that presents with a sharp bend in its proximal or middle part, folding back on itself, and may present with secondary lobes [13].

## 3. Left Atrial Appendage Physiology

The anatomy of the left atrial appendage facilitates its function as a receptacle during left ventricular systole, a blood drain from the pulmonary veins to the left ventricle in early diastole, a contractile room that assists in late diastolic left ventricular filling, and an early systolic suction source [14]. Early studies have also discovered its endocrine function as it contains the greatest density of atrial natriuretic factor (ANF) granules in the left atrium, which approximates 30% of all cardiac ANF [15]. Aside from these, the left atrial pressure helps in maintaining the LAA through the presence of stretch-sensitive receptors that can regulate the heart rate. Kappagoda et al. [10] found that distension of the LAA via fluid infusion led to diuresis, an increased Na(+) excretion rate, and an increased heart rate [16].

The LAA has an initial passive phase of emptying in protodiastole and another active phase of emptying during left atrial contraction and a monophasic pattern of filling [17]. During sinus rhythm, a washout from the LAA prevents blood from pooling and stagnating. After the mitral valve opening, the normal flow cycle begins with an early diastolic forward flow or LAA emptying, determined by the intracavitary suction via ventricular filling [18]. The cycle initializes with a phase of forward flow out of the appendage after the start of transmitral flow in early diastole, followed by a short backward flow into the appendage. The forward flow phase is fixed to the start of early diastole, suggesting a causal relation between left ventricular relaxation and early appendageal emptying [10]. LAA filling occurs after the LAA contraction and is a result of the combined effects of elastic recoil and LAA relaxation. This is followed by systolic reflection waves, which are low-velocity, multiple, alternate inflow–outflow, and usually seen in bradycardic patients [19].

## 4. Pathological Role in Thrombus Formation

It is when disruptions in this normal flow happen that pathology arises. The flow pattern in patients with atrial fibrillation is marked by a rapid alternation of emptying and filling, with lower velocities [17]. Furthermore, the flow pattern in atrial fibrillation patients can be characterized either as a saw-tooth emptying pattern or one without any active emptying pattern that is linked to the highest incidence of spontaneous echo contrast and thrombus [20].

Zabalgoitia et al. associated LAA peak flow velocities of ≤20 cm/s (RR 1.7, *p* = 0.008), disruptions in LAA peak flow velocities, and fractional area change with the occurrence of thrombus formation secondary to blood stasis. Compared to low-risk individuals, atrial fibrillation patients with hypertension (moderate risk) are at risk of atrial appendage thrombi (RR 2.6, *p* < 0.001) and reduced flow velocity (RR 1.8, *p* = 0.003) [21]. A study by Takada et al. which found low flow velocity as a predisposing factor for stroke among non-valvular atrial fibrillation patients without other sources of emboli further supports this [22].

Non-treatment and even undertreatment with oral anticoagulation of patients with atrial fibrillation resulted in a high incidence of stroke [23]. The CHA_2_DS_2_-VASc score was created to assess the risk of stroke or other thromboembolic events among non-anticoagulated patients with non-valvular atrial fibrillation. It uses clinical parameters (i.e., age, sex) and clinical history of diabetes, hypertension, congestive heart failure, thromboembolism, and vascular disease to risk stratify non-valvular atrial fibrillation patients as low- (0), intermediate- (1), and high-risk (≥2) [24].

## 5. Assessment and Imaging

**Figure 1:**
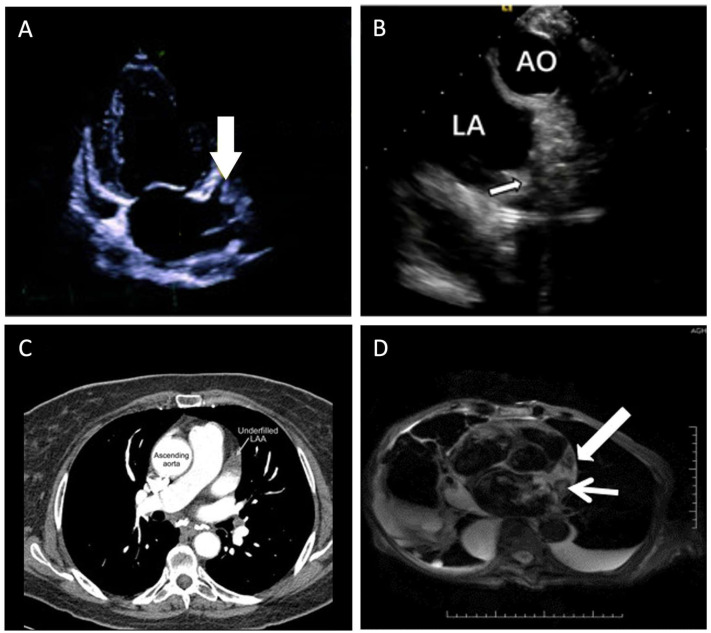
Imaging modalities to evaluate the left atrial appendage. (**A**) Transesophageal echocardiography—apical two chamber view during transthoracic echocardiogram without contrast showing thrombus in LAA [25]. (**B**) Intracardiac echocardiography—view from right ventricular outflow tract [26]. (**C**) Multi-detector computed tomography—axial image using MDCT showing underfilled or “abnormal” LAA with failure of contrast to fill the LAA (arrow) [27]. (**D**) Cardiac magnetic resonance—CMR paraxial view demonstrating T2-weighted image of the LA and LAA with fresh clot (narrow arrow) showing higher signal intensity than the old clot with reduced central signal intensity (broad arrow) [28]. *AO—aorta, LA—left atrium, LAA—left atrial appendage, MDCT—multi-detector computed tomography, CMR—cardiac magnetic resonance*.

Currently, the modality of choice for evaluating LAA is transesophageal echocardiography (TEE). Other non-invasive imaging modalities such as cardiac magnetic resonance (CMR), multi-detector computed tomography (MDCT), and intracardiac echocardiography (ICE) can also be used. The different imaging modalities can be found in Figure 1.

### 5.1. Echocardiography

Transesophageal echocardiography is the main echocardiographic modality used in assessing the LAA. Other types include intracardiac echocardiography and Doppler echocardiography. TEE has high sensitivity and specificity (close to 100%) in assessing the presence of atrial thrombi [29]. The use of TEE to exclude LAA thrombi allows safe and early cardioversion, preventing the need for extended anticoagulation therapy prior to cardioversion [30]. Ultrasound contrast agents are used to enhance the visualization of the LAA [13] and can demonstrate filling defects in the LAA [31,32]. A better frame rate of TEE results in higher-resolution images but is limited by inadequate imaging planes.

ICE is a less sensitive alternative in the absence of TEE [33]. Multiple views and detailed imaging of the LAA can be provided to diagnose the presence of thrombi [34,35]. ICE is also helpful in evaluating the LAA anatomy and dimensions to guide device placement and selection. Moreover, it is helpful in imaging the fossa ovalis to guide transseptal puncture and verifying LAA occlusion devices’ efficacy and stability [36,37]. The main limitations of ICE are the cost and lack of multiplanar capabilities and, as a result, it only provides suboptimal imaging of the LAA. 

Doppler echocardiography is often used for functional evaluation of the LAA to better assess the LAA and the risk of thromboembolism [38]. To exclude LAA thrombi, evaluation of the LAA’s Doppler velocities is essential. Color flow Doppler imaging can show areas with absent or decreased color flow within the appendage, which may indicate the presence of thrombi [39]. Furthermore, a color Doppler assessment of the LAA flow signals is acquired from an LAA long-axis view (between 60 and 90 degrees), and then it is sampled at the site of maximum flow velocity, which is determined by the color flow imaging scale (normally at the proximal third or mouth of the appendage). This maintains an optimal parallel angle with the flow and averaging of different cardiac cycles [39].

### 5.2. Multi-Detector Computed Tomography

Multi-detector computed tomography (MDCT) produces 3D volumetric data of the whole heart, which can be reconstructed along different cardiac phases and planes to give an accurate evaluation of the LAA anatomy. Recent advances in MDCT now allow 3D imaging, high spatial and temporal resolution, and quantitative assessment to allow successful identification of LAA thrombi and non-dense clearing spontaneous echocardiographic contrast (SEC), as shown using TEE [40,41,42,43]. An MDCT scan that is positive is not very specific to the presence of a thrombus. Hence, the high rate of false-positive test results and poor interobserver variability are the main limitations for precise detection of thrombi using MDCT [44]. Other limitations of MDCT include the use of significantly higher radiation doses, iodine-based contrast media use, and lower temporal resolution than TEE [45].

### 5.3. Cardiac Magnetic Resonance (CMR)

CMR is a non-invasive imaging modality, which is used as an alternative for those cases in which TEE is not feasible, such as in patients with unsuccessful TEE probe insertion. This imaging modality visualizes the LAA size and function accurately, and can also detect thrombi in patients with atrial fibrillation [46]. Tissue characterization can be facilitated non-invasively with the ability to differentiate between fresh (increased signal intensity) and old (decreased signal intensity) thrombi. Compared to TEE, CMR imaging has been shown to be good at detecting thrombi, although with an overestimation of thrombi size [47]. The disadvantages of CMR include increased cost, lower spatial resolution, the increased time duration of study, dependence on breath holds, the risks reported with gadolinium-based contrast agents, the presence of certain devices precluded from CMR imaging, and its inability to be performed in patients with implanted cardiac devices [48].

## 6. Current Clinical Approach

The left atrial appendage poses significant risk for thromboembolism in patients with atrial fibrillation [49]. Treatment for atrial fibrillation includes rate and rhythm control, anticoagulation therapy, cardioversion, ablation, and more recently, closure, exclusion, or excision of the left atrial appendage [50]. This section discusses the currently available options for thromboembolism prevention using LAA closure, exclusion, and excision. 

### 6.1. Indications for Left Appendage Occlusion (LAAO) 

The EHRA/European Association of Percutaneous Cardiovascular Interventions expert consensus statement on catheter-based LAAO has identified five patient categories in whom LAAO should be considered with regard to its risks and benefits. Clinically, the most widely recognized indication for LAAO is stroke prevention in patients at high thromboembolic risk (CHA_2_DS_2_-VASc ≥ 2) and with contraindications to oral anticoagulants (OACs) due to a history of significant bleeding like intracranial bleeding [51]. 

Another indication is stroke prevention in high-thromboembolic-risk patients (CHA_2_DS_2_-VASc ≥ 2) and the increased bleeding risk associated with systemic OACs in three patient groups: (1) patients with a HAS-BLED score ≥3, (2) patients requiring the prolonged period of triple anticoagulant and antiplatelet therapy for severe coronary artery disease treated with stents, and (3) patients with end-stage renal dysfunction (creatinine clearance 15–30 mL/min). The decision to implant LAAO is still an individualized risk–benefit evaluation with novel oral anticoagulants (NOACs) being still the main strategy of choice [51].

LAAO is potentially indicated in using the device as an alternative to OACs in patients who are eligible for OACs in whom there is no increased risk of bleeding, which only represents a small minority of current LAAO procedures [52].

The last indication according to the EHRA consensus on potential indication for LAAO is in patients at high thromboembolic risk (CHA_2_DS_2_-VASc ≥ 2) undergoing pulmonary vein isolation that are planning to discontinue OAC use after ablation [53].

### 6.2. Devices

**Figure 2:**
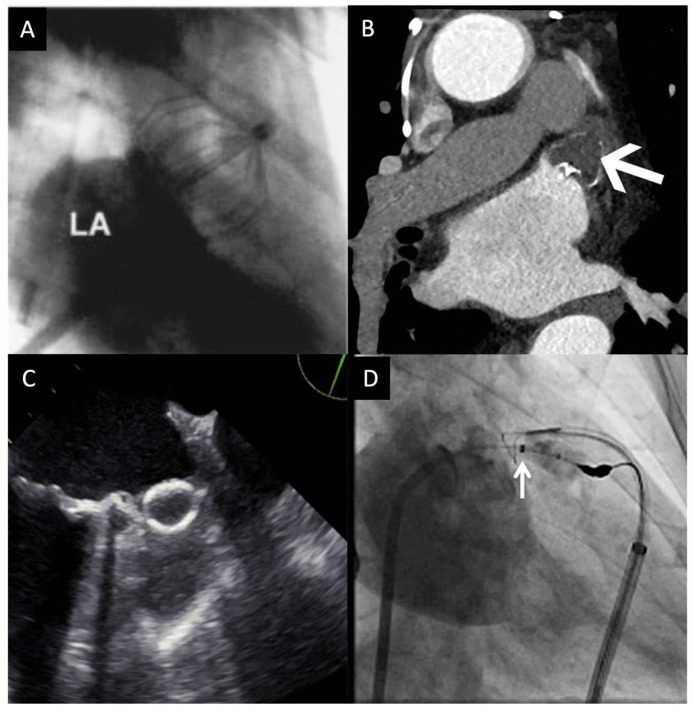
Devices used for left atrial appendage occlusion. (**A**) Percutaneous left atrial appendage transcatheter occlusion system [55]. (**B**) WATCHMAN [56]. (**C**) Amulet [57]. (**D**) Lariat [58]. *LA–left atrium*.

The currently approved devices for LAAO include WATCHMAN and Amplatzer Amulet. Patients who undergo left atrial appendage occlusion need to be suitable for short-term anticoagulation therapy, as the post-procedural guidelines indicate warfarin and anti-platelet therapy in the short term or indefinitely [54]. Percutaneous left atrial appendage occlusion has shown that it can be effective in reducing thromboembolic events in patients with an aversion to oral anticoagulation therapy. The devices used for the procedure are shown in Figure 2. 

#### 6.2.1. Percutaneous Left Atrial Appendage Transcatheter Occlusion (PLAATO) System

PLAATO was the first percutaneous device used to close the left atrial appendage [59]. The device consists of a self-expanding nitinol cage that is placed proximally in the orifice of the LAA. This functionally removes the LAA, preventing blood from entering or escaping. Multiple studies indicated a high success rate (90%) of occlusion and reduced risk of stroke (2.3% vs. expected 6.6%) [60]. In the PLAATO study, two patients died in the first 24 hours after the procedure (1.1%) and seven patients had serious adverse events (3.9%) (cardiac tamponade or device embolization) [52]. A meta-analysis showed that when compared to other transcatheter left atrial occlusion device, PLAATO had the highest all-cause mortality and cardiac/neurological mortality [61]. In 2007, the device was discontinued and replaced with the WATCHMAN device [59].

#### 6.2.2. WATCHMAN

The WATCHMAN device was approved for LAAO by the FDA in 2007. Much like the PLAATO device, it is a self-expanding nitinol cage. Barbs are used for device fixation. A permeable polyester mesh covers one end of the implant [59]. Based on the National Cardiovascular Data Registry Left Atrial Appendage Occlusion Registry, the most common procedural indications for WATCHMAN implantation were increased thromboembolic risk, history of major bleeding, and high fall risk with the majority of patients having multiple procedural indications [62].

Multiple randomized control studies have indicated that the WATCHMAN device is non-inferior to warfarin therapy for patients with increased risk of clotting [51,63,64]. Furthermore, a recent meta-analysis of three randomized control trials indicated that WATCHMAN has a reduced risk of hemorrhagic stroke (RR: 0.22; 95% CI: 0.08 to 0.58; *p* = 0.002), cardiovascular mortality (RR: 0.65; 95% CI: 0.44 to 0.95; *p* = 0.03), all-cause mortality (RR: 0.78; 95% CI: 0.62 to 0.99; *p* = 0.04), and non-procedure-related major bleeding (RR: 0.53; 95% CI: 0.38 to 0.74; *p* = 0.0002) when compared to OACs [65].

#### 6.2.3. Amplatzer Amulet 

Amplatzer Amulet was approved for LAAO in 2021. It consists of a braided nitinol mesh disk and lobe connected by a waist [66]. Clinical trials have demonstrated non-inferiority to traditional warfarin therapy for patients with atrial fibrillation who are at high risk of stroke or systemic embolism [66]. Furthermore, when compared to the WATCHMAN device, LAA closure with Amplatzer Amulet was non-inferior with a high success rate at 45-day follow-up [66]. Amplatzer Amulet demonstrated more procedure-related complications (4.5% versus 2.5%); however, major bleeding and all-cause mortality were similar between the two devices (10.6% vs. 10.0% and 3.9% vs. 5.1%, respectively). Amplatzer Amulet also demonstrated a higher rate of successful occlusion of the LAA compared to WATCHMAN (95% CI, 0.41–3.66; *p* < 0.001 for noninferiority; *p* = 0.003 for superiority) [67].

#### 6.2.4. Lariat 

The Lariat system is a combination of percutaneous and epicardial LAA closure using a soft tissue snare. Via percutaneous access, a magnet-tipped catheter is placed in the LAA as a guide for the snare that is delivered via the pericardium. Currently, this device has 510 K clearance from the FDA [58]. Despite not having full FDA approval, the Lariat device is a popular option (off-label) for atrial fibrillation patients with contraindication to oral anticoagulation. Many studies have indicated a relatively high success rate of LAA closure (86%) [68]. In one study, Lariat showed a reduced incidence of LAA leak at one-year follow-up compared to WATCHMAN (14% vs. 21%; *p* = 0.019) [69]. Multiple studies have shown high post-procedural complication rates; however, they appear to be comparable to WATCHMAN (2.7% vs. 2.7%) [68]. The clinical outcomes of Lariat and other devices are summarized in Table 1.

### 6.3. Surgical LAAO

It is common for patients with atrial fibrillation undergoing cardiac surgery to receive concomitant LAA obliteration. The techniques for obliteration include excision, stapler removal, running sutures, and the use of specialized devices such as AtriClip^®^. Individual techniques have been well described elsewhere, so here we will discuss surgical obliteration as a whole. 

A recent meta-analysis by Tsai et al. concluded that when compared to patients who underwent cardiac surgery (either valve surgery or CABG), those who received LAAO concomitantly had a significantly reduced risk of stroke and all-cause-mortality, without increased risk of bleeding [70]. Another meta-analysis by Homamed et al. concluded that LAAO provides a significant reduction in thromboembolic events, but no significant change in all-cause mortality, major bleeding, or myocardial infarction [71]. A large study by Melduni et al. (*n* = 10,633) suggested that those who underwent surgical LAAO were more likely to have early post-operative atrial fibrillation than those who did not undergo LAAO, without significant change in risk of stroke or mortality [72]. A study by Yao et al. reports that those who undergo LAAO have more healthcare appointments related to atrial fibrillation and more hospitalizations compared to those who do not undergo LAAO [27]. A randomized controlled trial comparing LAAO with anticoagulation to anticoagulation alone found that LAAO recipients had a reduced risk of stroke, but no change in perioperative bleeding, heart failure, or death [73]. There have been no studies comparing surgical LAAO to percutaneous LAAO. 

### 6.4. Risks and Disadvantages of Left Atrial Appendage Closure

Whether carried out percutaneously or surgically, left atrial appendage closure is not without risk. The major risks for percutaneous procedure include cardiac effusion/tamponade, stroke, and device-associated thrombi [74]. Based on the most recent WATCHMAN clinical trials and registries, the cardiac tamponade incidence is limited to 1.3 percent [75,76]. Freeman et al. reported hemorrhagic stroke in 0.01 percent and ischemic stroke in 0.12 percent based on the National Cardiovascular Data LAAO Registry [77]. Aside from these, further catastrophic complications that may occur intraoperatively or post-operatively include device embolization in the left atrial cavity, left ventricle, and aorta [78]. Real-world data from the National Cardiovascular Data LAAO Registry reported a 0.07 percent incidence [77].

A study by Simard et al. identified the following as risk factors for device-associated thrombi: hypercoagulability disorders, renal impairment, pericardial effusion, implantation depth >10 mm from the pulmonary vein limbus, and non-paroxysmal AF [79]. Moreover, one study found that the risk factors associated with incomplete closure include female sex, hyperlipidemia, and hypertension [80]. Darden et al. found females to have higher risk of in-hospital adverse events relative to males, such as major bleeding (1.7 versus 0.8 percent) and pericardial effusion (1.2 versus 0.5 percent) [81].

Incomplete closure of the LAA is not uncommon with a surgical approach. This results in a patent communication, which only exacerbates the risk of stroke. In addition to this, the left circumflex artery is often proximal to the site of obliteration, necessitating mastery of surgical anatomy and skills.

### 6.5. LAA Occlusion Outcomes

The clinical results of LAA occlusion in comparison to oral anticoagulation were explored in multiple studies. Table 2 shows the outcomes of LAA occlusion in comparison to oral anticoagulation in previous studies from 2001 to the present.

In the LAAOS III study, which first explored the prevention of ischemic stroke or systemic embolism in patients who had LAAO coupled with usual post-operative care, including oral anticoagulants, systemic embolism or ischemic stroke occurred in 114 participants (4.8%) in the LAAO group and 116 participants (7.0%) in the non-occlusion group. This showed a significant reduction in risk of ischemic stroke or systemic embolism (HR 0.67, 95% CI 0.53–0.85, *p* = 0.001) [73].

It is noteworthy to highlight multiple randomized controlled trials, including PREVAIL, PROTECT AF, and PRAGUE 17, which have shown the efficacy and safety of LAAO in comparison to medical management [52]. The PROTECT AF trial demonstrated non-inferiority of LAAO versus warfarin in both primary efficacy rate and primary safety events. The primary efficacy rate (composite endpoint of stroke, cardiovascular death, and systemic embolism) of the LAAO group was 3.0 per 100 patient-years (95% credible interval [CrI] 1.9–4.5) compared to 4.9 per 100 patient-years (2.8–7.1) in the warfarin group (rate ratio [RR] 0.62, 95% CrI 0.35–1.25). Similarly, the primary safety events were more frequent in the LAAO group than the warfarin group (7.4 per 100 patient- years, 95% CrI 5.5–9.7, versus 4.4 per 100 patient-years, 95% CrI 2.5–6.7; RR 1·69, 1.01–3.19) [52].

In the PREVAIL trial, stroke or death at 18 months was found to occur in 0.064 in the LAAO group compared to 0.063 in the warfarin group, which did not reach the pre-established non-inferiority criteria for the trial [73]. The rate for stroke or systemic embolism >7 days post-randomization was 0.0253 in the LAAO group compared to 0.0200 in the warfarin group (risk difference 0.0053 [95% CrI: −0.0190–0.0273]), which achieved non-inferiority [73]. 

The PRAGUE17 trial showed that LAAO was non-inferior to NOAC. The Amplatzer Amulet or WATCHMAN device was used for the LAAO, while the control group was given antiplatelet therapy for 3 months. The primary clinical findings showed that the net outcome of CV death, stroke, TIA, systemic embolism, etc., was similar in both treatment groups (hazard ratio [HR] 0.84, *p* value for non-inferiority = 0.004) [82].

After 3.8 years of follow-up, the combined outcome of preventing stroke, systemic embolism, and cardiovascular death among LAAO patients was shown to be non-inferior and superior to those of warfarin patients in the PROTECT AF trial, 8.4% vs. 13.9%, respectively (rate ratio [RR] 0.60, 95% credible interval, 0.41–1.05). There was reduced cardiovascular mortality (3.7% vs. 9.0%; hazard ratio [HR], 0.40; 95% CI, 0.21–0.75; *p* = 0.005) and all-cause mortality (12.3% vs. 18.0%; HR, 0.66; 95% CI, 0.45–0.98; *p* = 0.04) [83].

**Table 2:** Clinical studies on left atrial appendage occlusion outcomes.

Author	Year	Multicenter	Study Type	Findings
Healey, J. [84]	2005	No	Randomized Controlled Trial	At the time of CABG, LAA occlusion is safe. The rate of complete occlusion improves to acceptable levels with the use of stapling devices and more experience.
Whitlock, R.P. [85]	2013	Yes	Cross-Sectional Study	This study demonstrated that LAA occlusion could be safely performed at the time of cardiac surgery.
Holmes, D. [63]	2014	Yes	Randomized Controlled Trial	This study showed that LAA occlusion was non-inferior to warfarin for systemic embolism >7 days post-procedure or ischemic stroke prevention. Furthermore, this study supported that LAA occlusion is a better alternative to warfarin therapy for stroke prevention in patients with non-valvular atrial fibrillation who are without absolute contraindication to short-term warfarin therapy.
Belgaid, D. [86]	2016	Yes	Randomized Controlled Trial	This study exhibited that LAA occlusion is a good alternative to chronic warfarin therapy for stroke prevention among patients with atrial fibrillation.
Zhou, X. [87]	2016		Systematic Review	In stroke reduction, left atrial occlusion with the WATCHMAN device had the same effectivity as compared with warfarin. Compared to warfarin, surgical LAAO had positive outcomes but the evidence was less powerful due to the small sample size.
Nielsen-Kudsk, J.E. [88]	2017	Yes	Observational Propensity Score-matched Study	In atrial fibrillation patients having sustained an ICH, LAAO was suggested to be of major clinical benefit.
Godino, C. [89]	2020	No	Observational Prospective Study	NOACs and LAAO has similar performance in terms of major bleeding and thromboembolic events up to two-year follow-up in non-valvular atrial fibrillation patients at high bleeding risk.
Ding, W.Y. [90]	2022	Yes	Cohort Study	For stroke prevention in patients with atrial fibrillation, LAA occlusion was found to be a suitable alternative to NAOC therapy.
Korsholm, K. [91]	2022	No	Cohort Study	In comparison with DOAC, LAAO therapy showed lower risk of the composite outcome of major bleeding, stroke, and all-cause mortality in patients with atrial fibrillation and prior stroke.
Nielsen-Kudsk, J.E. [92]	2021	Yes	Cohort Study	LAAO have lower risk of major bleeding and mortality as compared to DOACs among high-risk atrial fibrillation patients while keeping similar stroke prevention efficacy.
Whitlock, R.P. [73]	2021	Yes	Randomized Controlled Trial	This study found that the risk of systemic embolism or ischemic stroke was lower with accompanying LAA occlusion performed during surgery than without it.
Noseworthy, P. [93]	2022	Yes	Cohort Study	When compared with non-vitamin k antagonist oral anticoagulants, LAAO was linked with a lower risk of mortality and no significant difference in the composite outcome risk and this showed that LAAO might be a better option in select patients with atrial fibrillation. It was observed that there was higher bleeding risk associated with LAAO, and because of this, there was need to optimize systemic efforts and post-procedural antithrombotic regimens to assess and address bleeding predispositions.

## 7. Conclusions

The LAA is clinically important because it is a major site of thrombus formation in atrial fibrillation and mitral valve disease. Due to the LAA’s properties and relations, it may be utilized as a decompression chamber in cases where left atrial pressure is increased. The exact pathogenesis of thrombus formation is not yet known, but it is hypothesized that the stasis of blood flow within the LAA is a key factor.

Occlusion of the LAA is a good alternative to warfarin for stroke prophylaxis. However, it will not prevent every episode of thromboembolism, especially in patients with mitral valve disease. This is important because patients with left ventricular failure and valvular disease may be more affected.

Alternative treatments need to be explored to further understand how to create more effective outcomes for thromboprophylaxis in patients with atrial fibrillation. This will help determine which course of treatment should be selected, as well as how to manage significant adverse effects from the specific treatments.

## Figures and Tables

**Table 1:** Left atrial appendage occlusion devices. PLAATO [59], WATCHMAN [62], Amplatzer Amulet [58], and Lariat [66].

Device ^a^	PLAATO [59]	Watchman [62]	Amplatzer Amulet [58]	Lariat [66]
Success Rate of Implantation	90.0%	95.1%	98.4%	86.0%
Complication Rate	Not assessed ^b^	2.2%	4.5%	10.0%
Stroke	2.3%	2.3%	2.8%	1.0%
Bleeding	3.3%	0.8%	10.6%	9.0%
Mortality	Not assessed ^b^	2.6%	3.9%	0.14%

^a^ Outcomes cited are from different studies. Definitions of each outcome may vary from one study to another. ^b^ Study discontinued at 5-year follow-up due to financial constraints.

## Data Availability

Not applicable.
